
JOURNAL INFORMATION
==============================
NLM Title Abbreviation: Med Chir Rev
NLM Journal ID: 0034617

The Medico-Chirurgical Review

Publisher: Biomedical Journal Digitization Project

ARTICLE INFORMATION
==============================
PMCID: PMC4966173
PMID: 29917596
Article version: 1
Subjects: Reviews

Dr. Macculloch on the Diseases Produced by Malaria

Print publication date: 1828 Jul 1
Volume: 9
Issue: 17
First page: 49
Last page: 67
1828 May 10
License: This work is free of known copyright restrictions. For more information, please see PMC Back Issue Digitization.
License URL: https://creativecommons.org/publicdomain/mark/1.0/

==============================

### VI.

*An Essay on the Remittent and Intermittent Diseases, including, generically Marsh Fever and Neuralgia; comprising, under the former, various Anomalies, Obscurities, and Consequences; and, under a new Systematic View of the latter, treating of Tic Douloureux, Sciatica, Headach, Ophthalmia, Toothach, Palsy, and many other Modes and Consequences of this Generic Disease.* By JOHN MACCULLOCH, M.D. F.R.S. &c. &c. Physician in ordinary to His Royal Highness Prince Leopold of Saxe Cobourg. In two Volumes, 8vo. 1828.

IN some late Numbers of this Journal, we have given a very full account of Dr. Macculloch's work on MALARIA, as the cause of the various (at least variously denominated) diseases in the two volumes now before us. It will require three or four articles to convey any thing like an analytical delineation of these volumes; for, although it will be readily perceived that our learned author has a hobby-horse, on which he has quietly rode for more than 20 years past, pursuing the bent of his own inclinations, investigations, and meditations, yet we do not think that his speculations are all visionary, though some of them are probably seen through a coloured, if not a distorting medium. Dr. Macculloch is a great philosopher and logician—and he wishes to see all medical inquiries carried on according to the strict rules of philosophy and logic. That he is frequently a little ruffled in his temper, on finding that physicians have not generally conducted their investigations on these *beau-ideal* models, will be sufficiently apparent as we proceed. That he is sometimes pretty sharply critical —we had almost said *cynical*, will probably be inferred from the following passage in the preface to these two volumes. After telling us that science begins with conjecture and assertion—that its infancy is the reign of the imagination—that "physic is yet in that very infancy, wandering about its own fairy land"—that this science abounds in bad observation, in imaginary experience, "and even in positive *mala fides*"—"that the laws of philosophy and *logic* (what will Sir Gilbert Blane say to this?) have scarcely yet found their way into it," our author winds up with this terrible Philippic.

"The language of truth is simple and brief, but that is not the language of physic. Its words have meanings, and the same words have always the same meaning: but this is not the language of physic. The language of error is multitudinous, variable, vague and unsteady: and this is the language of physic. If there be a philosophical reader who doubts this, if there be a logician, a man accustomed to evidence, who has not read medical books, let him read even the most celebrated, and be satisfied."

These are hard words master! Perhaps, if the learned author had toiled, during the said 20 years, at the bedside of sickness, he would have been more merciful to his brethren. It is not in books alone that we see the most clashing testimonies, contradictions, and contrarieties. He who has seen most of human nature, and especially of human maladies, is most convinced of the existence of these contrarieties, and of the impossibility, in the present state of our knowledge, of reconciling them by means of philosophy and logic. We make allowance, therefore, for Dr. Macculloch, because we believe he has been in the habit of viewing things rather as they *should be*, than as they *are*. The philosopher forms an imaginary picture, in his mind, of what the order of things ought to be in this world—but when, like Parnel's hermit, he comes out among men, he discovers a terrible difference between the closet and the crowd! The disappointment of the worthy Doctor, at not finding physicians to be philosophers, brings up the rather ludicrous reminiscence of Sir Joseph Banks' disappointment, at finding certain little animals remain black, after undergoing the severe discipline of ebullition,

"Fleas are not lobsters, d—— their souls."

Dr. M. observes that—"he who is now the philosopher in physic, is also a Pythagoras, while he does not *perceive* it." We confess that we cannot clearly *perceive* the drift of this occult passage—nor that of the following:—"The physician is like Nebuchadnezzar—he dreams, and death is the sentence of him who cannot divine what that dream was." We humbly propine that, when the physician dreams, he also sleeps—and we have a notion, that DEATH is not always most active when the doctor nods. But, leaving all prefatory matters aside, we come to the philosophy and logic of the work. The first of these two volumes is entirely occupied with the subjects of remittent and intermittent fevers—dysentery—and cholera. The first of these topics will afford ample materials for this article.

### ORDINARY REMITTENT OR MARSH FEVER.

Dr. M. must be an extremely discontented man. He sets out by informing us that, on no disease in the whole circle, has he derived less information from books than on this. "He must labour with no small discrimination, who would, from medical works, extract any rational account of the immediate causes of the disease, or of the real condition of the system under it—who would discover any intelligible and consistent method of cure—who would even be always certain that it is of this disease, and not of contagious fever, that he is reading." What if we were to tell Dr. M., that the causes of this fever are not always precisely of the same kind—and that this observation might apply to the conditions of the system—and, consequently, to the methods of cure! Nay, we would have little hesitation in going a step farther, and saying, that Dr. M. himself would not always be able to discriminate between marsh fever, under particular circumstances, and contagious fever, if it do not actually take on a contagious character.

The object of our author, in this Essay, is to class together some disorders which appear to him to have been misunderstood, as well as misplaced, in relation to their causes and characters—and, consequently, whose treatment has been erroneous. At the head of this class, as the offspring of malaria, stands remittent fever—the most important link in the philosophical chain—and the cause of many other diseases. Dr. M. properly observes, that the more perfect forms of diseases, as described in books, are much more rare, in nature, than the ill-defined forms, and, hence, the unreasoning, and, indeed, the inexperienced practitioner, is daily at a loss to name or classify the multitudinous forms that present themselves to his view. It is our author's object, therefore, in this work, chiefly to notice that which is obscure in itself, or least generally known, in marsh fever; thus making the Essay a kind of supplement or addition to the systematic descriptions already existing.

Although there can be no reasonable doubts, that the general cause of remittent fever, in its perfect form, is the application of malaria; yet our author acknowledges that various other causes do produce it, as heat, errors of diet, fatigue, cold, mental anxiety, &c. "But every one of these is an accessory cause of many other disorders;" and if malaria be more copiously generated, or more widely diffused, than is usually suspected, it may still be the real productive cause, where the causes above-mentioned are only auxiliaries, though the only ones that are apparent. The subject of the malaria itself has been amply discussed in a former volume, and all that he can permit himself here to say is—"that he has attempted to prove that all the fevers of any moment, which are not produced by contagion, are the effects of malaria, very often, or, perhaps, very generally, overlooked." All other fevers, arising from other causes than malaria and contagion, are comparatively trifling in number or power. Our author recognizes one exception to the rule respecting the cause of remittent fevers—namely, HABIT. It is well known that agues and remittents are sometimes reproduced, where the original cause cannot be in operation. It is probable, however, that, in all these relapses, some of the auxiliary causes have been exerting their influence on the constitution.

After some observations on the comparative susceptibilities of natives and strangers, in malarious countries, Dr. M. touches on the *time* which intervenes between the reception of the poison and the manifestation of its effects.

"If my own frequent observations shew that fever may be induced within half an hour after exposure to malaria, and that a single inspiration, or the space of a very few seconds, is amply sufficient for the purpose, this is also an opinion most decidedly stated by many French and Italian physicians whose experience and acuteness will not be questioned. It is equally the opinion of other observers, not physicians, and, therefore, without the bias which might be suspected in such cases: of military, and chiefly of naval men, whose observations have been founded on the momentary and transitory effects of a breeze of wind, and especially of a land wind blowing off to sea. In France and in Italy, to confirm this, instances are known and recorded, of labourers dying instantaneously from merely sitting or lying down on the ground, and of others who from looking into a ditch or drain, have been struck dead by that poison which, of course in a minor degree, would have merely produced a fever. Lind, also, whose authority stands high, describes the instant seizure with nausea and delirium, as many others have done; so that respecting this part of the question there needs be no dispute."

That the miasmal poison may, in certain states of uncommon concentration, be capable of producing an instantaneous, or even fatal effect on the nervous system, we do not deny; but that a regular fever is so quickly produced, we are inclined to doubt. It seems, in general, to require a certain period for concoction in the system, before fever is evolved. The extreme length of the interval next engages our author's attention, and after a minute examination of evidence, he appears to conclude, that 15 or 20 days form the utmost limit—perhaps more than the limit. Thus a ship's company became affected with remittent fever, on the coast of Africa—the ship put to sea, but some of the men continued to fall ill with the fever till the 20th day, after which no more cases occurred. Here, too, Dr. M. says there may have been fallacy. There may have existed a foul hold, from whence the febrific miasm continued to ascend—or the fever might have become contagious. "A remittent will become, or perhaps produce, in any given individual, a contagious typhus, under confinement." But independent of this hybrid, or contingent disease, we think there are sufficient facts on record to prove, that miasmal fever may take place after a longer absence from the source of the miasm.

"Many physicians or surgeons, both English and French, have said, that even after six months, many soldiers who had been at Walcheren and had escaped the fever there, were seized with the same disease in other countries; asserting also, that the poison had remained during that time dormant in the constitution. Pym is one of those who thinks thus, and so I imagine does Blane; while Bancroft believes, that the intermittents of spring are the produce of malaria received in the previous autumn. Baumes, resembling Lind, limits the term to fourteen days; but Ferrus, coinciding with the former, relates in proof, a case of a soldier who having escaped at Walcheren, was affected with this fever six months after, on the Niemen; as there is also a case quoted in evidence, where an English regiment became attacked in the same circumstances in England, after eight months."

An objection might be readily raised to the latter fact, that a fever produced *on* the banks of the Niemen, may be produced *by* the banks of the same river. And so an English regiment serving in Spain, after the expedition to Walcheren, might very readily pick up some malaria in the former place, without any necessity for carrying it from the banks of the Scheldt. In a physiological point of view, it is difficult to conceive how a poison of this, or of any other nature, should lie dormant so long in the system. It is easier to conceive the reception of a new poison, and the production of a new disease.

Dr. M. very properly passes over, by a reference to a host of authors, the symptoms of remittent fever, both in its simple and complicated forms—together with the almost endless modifications produced by climate, epidemic influence, and various other causes, including differences in the miasma itself.

"But I may mention, that while an undue, and apparently a morbid secretion of bile is the most conspicuous and common local affection, producing sometimes what is emphatically called the yellow fever, so the brain and other organs, and above all, the stomach and the bowels, are often found affected by inflammations, modifying materially the symptoms, and also demanding important modifications in the practice."

In referring to systematic writers for general descriptions and local peculiarities, Dr. M. *feels* himself compelled to notice that characteristic symptom, whence the disease derives its *name*. This is the remission or diminution of the intensity of the febrile symptoms, occurring once in the 24 hours, however variable in the period of its arrival, its duration, and degree. Sometimes this remission is so slight as not to be perceived, though errors are often committed here, especially when the remission occurs in the night, or in the absence of the medical practitioner. For our own parts, we can hardly say, that we have ever seen a decidedly continuous fever. There is an evening exacerbation and morning remission in almost every case. It is hardly necessary to say, that it is by a gradual prolongation of this interval of remission, that the so called continued fever of malaria becomes an intermittent—and this leads our author to speak of the terminations of the disease. There is, no doubt, a disposition in most fevers to resolve on certain days termed critical, though these are now but little observed.

"Three weeks may probably, however, include the much greater number of terminations in recovery, when the disease submits to the law of the critical days; while it is not uncommonly protracted to six weeks; and even in cases where its extreme mildness might have led us to expect an earlier solution."

Where this law of crisis does not seem to exist, we can assign no period for the recovery or the fatal termination. The favourable termination is often perfect, in all climates; but it not unfrequently changes to an intermittent, easily, in general, removed by proper remedies—but often peculiarly inveterate and indomitable, as those who have witnessed Walcheren, some parts of China, Greece, Italy, Spain, Moldavia, and many other countries can testify.

But remittent fever not only terminates in ague—it frequently produces, or ends in, "the local and painful affections of the nerves which may be ranked under the general term Neuralgia." After alluding to the production of "paralytic affections," by remittent fever—or the termination of this fever in such affections, Dr. M. makes the following observations, which bear on a point of pathology, or etiology, recently mooted in one of our medical societies.

"That marsh fever does act directly, itself, or its generating poison, on the nervous system, is proved by the state of apoplexy or profound coma with which the attack is sometimes ushered in; a fact common in Italy, and known by the name of febbre larvata; though, in this case, mere intermittent may also be the supervening disease, instead of remittent. That, in these fevers, the affections of this nature have been attributed to local diseases of the brain, I know; and such events may doubtless occur. But this does not explain the cases in question, where the affection of the brain is instantaneous, following directly the application of the poison, even before fever is produced, and resembling that which occurs from the application of other poisons, whether to the lungs or the stomach.

"Farther, as it is the effect of Malaria to produce the local affections of particular and single nerves, either with supervening or present palsy, or without either, while the brain is not affected, and while no local inflammation or other disease of that organ can be supposed to exist, from there having been no previous fever, it is plain that Malaria does exert a power of some kind on the nervous system directly; on the whole, or on more or fewer of its parts, even to a single point in the minutest nerve."

Thus the larger paralytic affections, as hemiplegia, or paralysis of a leg or arm, consequences of an intermittent or remittent, may, he thinks, result from "direct action on the nervous system," and, in this way, he also thinks, may be explained that diminution of intellectual power, proceeding often to perfect idiotism, which sometimes follows long-continued intermittents. Dr. M. admits, however, (what we think is the more probable solution) that there may, in such cases, be organic diseases in the brain itself, the produce of local inflammation there. He does not consider this, indeed, as the more common source of the paralytic or cerebral affection.

"But if malaria does produce direct apoplexy, as it also often brings on a comatose state which is exceedingly durable, both in remittent and intermittent, and if also it produces, not only local and similar effects on single nerves, but complete hemiplegia, it will be most necessary to inquire whether some of the cases of paraplegia or other palsy, especially as occurring in certain climates and in campaigns, are not instances of the same nature; since, whether our practice in such a disorder should be different or not from the treatment of palsy produced by simple cold, the philosophy of physic cannot fail to be improved by discovering causes and assigning distinctions."

The subject of visceral or glandular disease is next taken up. Most authors have looked upon these as the effects of the fever which preceded them—though a few writers have considered the visceral affection as the primary, and the fever as the secondary link in the morbid chain. Dr. M. appears to think that both hypotheses may be occasionally right. Thus, some physicians have maintained that the hepatitis of India is the direct effect of a morbific miasm—and, if so, other glands and organs may be the primary seat of action when malaria is received.

"Thus also, through France, Italy, Sicily, every where, it is common to find, not merely single instances, but a whole population, suffering from glandular diseases in their worst forms; while no fever is present, and while also, in many cases, it seems to be ascertained that no fever has preceded, or that there has at least been no severe remittent or intermittent as the cause."

Our author concludes this chapter with an expression of his disapprobation of those wild theorists who disbelieve in contagion altogether. No doubt the great mass of fevers in this country are devoid of contagious character, because comfort and cleanliness are observed—"but to assert that there is not such a thing as contagious fever, is to discredit evidence as numerous and incontrovertible as science or human affairs have ever produced." The fevers not contagious, he thinks, must fairly be ascribed to malaria.

The second chapter is on the chronic or relapsing, and obscure or anomalous remittent fever. This, Dr. M. observes, is a modification which is often but little noticed, while it is a source of great distress.

"If this peculiar variety is sometimes sufficiently severe and marked to be esteemed a fever, it is far from uncommon for it to be so slight as to pass for hectic, for what is called debility (a term without meaning) or for ill health, or delicate health, terms equally convenient to cloak ignorance; while not unfrequently also, it is characterized by the no less convenient phrase nervous, or even brings on the unlucky patient the charges of hypochondriasis or affectation."

To be more particular; there is, says Dr. M. a fever not uncommon among us, to which the popular name of nervous fever is not inaptly applied, when its symptoms are not severe. Dr. Cullen was unpardonable, he says, in confounding this with contagious fever, under the name of typhus mitior. It is not contagious, nor produced by contagion, as far as his own observations have gone. "Durability, or the property of prolongation, seems to be a peculiar character of marsh fever, under all its forms; and, until an unequivocal case of contagious fever, thus mild and thus durable, is produced, I must continue to believe, that all long-continued or often-relapsing fevers belong to the disorder under consideration." The same remarks apply to what Cullen denominates synocha and synochus. By this, however, Dr. M. does not mean to deny that there can be such a thing as a pure inflammatory fever, produced by cold, or the other causes usually assigned; but he believes that the disorder so called is very frequently "a fever of the remittent family, and produced by the same causes." Many plausible arguments are adduced in favour of this position, for which we must refer to the work itself. If this "low fever"—"fever on the spirits"—"fever on the nerves," (by all which names it is known) be not a modification of remittent, our author knows not how or where it is to be classed. Physicians must erect a new genus for it—since it does not belong to one or other of the two leading classes.

"And it is far from unimportant that this point should be clearly understood; as it is only thus that our practice can be justly regulated: while it is most certain, that by mistaking it for other diseases, the sufferings of the patients have often been, and are daily and everywhere, materially aggravated. And if the cause, the original one, be Malaria, as in the case of acknowledged remittent, whatever the causes of the relapses may be, we thus acquire the means of prevention; of which, as long as we mistake its nature, we cannot avail ourselves."

Among other arguments in support of the said position, Dr. M. adduces this one, that the disorder in question is among those habitual complaints included under the vague term "ILL HEALTH," which are the produce of low and wet situations, or of some of the soils formerly described as productive of malaria.

"Another argument is, that its relation to the marked or severe and terminating remittent, in slenderness of symptoms and in the frequency of its recurrence, is precisely that which intermittent, equally slender and equally returning, bears to a limited and severe intermitting fever, while I may lastly add, as a proof of its cause and return, that if it is especially subject to relapses in low and wet situations, as well as indebted for its very existence to those, so it is best cured, and especially when relapsing or repeated, or chronic, by change of air; that is, by change to a drier air as it is usually termed, or, what is the fact, by removal from its causes."

Dr. M. thinks it probable that the disease in question, is that which Dr. Haygarth has described, in the College Transactions, as inexplicable—"a peculiar state of permanent debility, enduring even for years, and without very marked disease of any kind." Unless it be attributable to the abuse of purgation and bleeding, Dr. M. is unable to account for this complaint, except by assigning some morbific exhalation from the earth as its cause. We must now allow our ingenious and indefatigable author an opportunity of describing the disease under consideration.

"This disorder may be found, and not unfrequently, with scarcely any marked symptom except mere muscular weakness; a debility on any attempt at exertion, which seems unaccountable, inasmuch as it occurs in persons, even in youth, and apparently strong, and is not very obviously accompanied by any proper febrile symptoms. At times, not even the appetite seems affected; and here, almost necessarily, the result is, to suspect the state of the patient's mind, or his moral dispositions, rather than his health; to suppose, for example, as I have often seen, that a soldier is 'shamming,' that an opulent female is indolent or affected, or a studious or professional man hypochondriacal.

"Yet, let an acute physician watch this disease, and he will be convinced that it is a disease, and moreover a fever. It commences and terminates like the remittent when best marked; and when it appears to be prolonged for months or years, as is sometimes the case, it will be easy to see that it has had intervals of cure, generally of self-cure, and relapse; and that, to each relapse, there is a period of weeks, not very uncommonly of six, while the intervals vary from one or two to any given number. Further, either the patient or the physician, or both, must be very inattentive if they do not discover that the paroxysm of extreme debility is fixed; that it is, in fact, a paroxysm, let its length be what it may, and that there is a diurnal period when it diminishes, or where the patient, who, possibly, could not stand, on getting up in the morning, is enabled to exert, and even to enjoy himself at night.

"Hence, as to some cases, at least, the truth of, as well as the reason for, a very common remark, that midnight is the nervous patient's holiday; though there are unquestionably many cases of nervous affection, and even of periodical returns and intermissions in this complicated class of disorders, which do not appertain to a remittent type of fever, or perhaps to any fever. The particular case here quoted, is one, of course, where the paroxysm attacks in the morning and the remission is at night; but while the periods are necessarily various, so are the results, as to the complaints, appearances, or sufferings of the patient. I shall presently trace some others of the more marked of these modifications."

Between the above and the unequivocal forms of remittent fever, there are, of course, a great number of shades, in which the obscure symptoms gradually multiply and become more conspicuous, indicating, as they advance, a more severe disease. It would not be difficult, Dr. M. adds, for any attentive physician, in tolerably extensive practice, "to collect a series of cases rising in exact gradation, from the simplest debility to the most perfect form of remittent fever." The appetite, though sometimes apparently unaffected, is generally irregular or capricious—vanishing in the paroxysms, and returning in the intervals. The tongue and the secretions will usually shew indications of disorder, but none peculiar or pathognomonic. These disorders of the primæ viæ Dr. M. looks upon, of course, as *effects*, though they are generally viewed as *causes* of disease.

"In the whole catalogue of ordinary practical errors, I know few indeed more common than that which views a sluggish state of the bowels as a primary disease, sometimes also a consequence of theoretic disorders of the liver, instead of considering it what it really often is, the produce of a febrile state, belonging, either to this fever or to some other initiative and similar cause. Nor is it difficult to account for this error, vulgar as it is common; since it is the consequence, partly, of seeing, in a disorder, nothing but obvious symptoms, and partly of that empirical practice for which England is so celebrated, and which, while it tends to blind the judgment, can, from its facility, be conducted by any one."

This will not be a very palatable doctrine to the routine practitioner, and especially to the disciples of Abernethy, who have had Halcyon days of it, for many years past, in consequence of the great simplicity, or rather uniformity to which practice was reduced, as a result of the doctrine that made but one cause for all disorders. We are disposed to think that the moral world will not *alone* suffer from those disturbances occasioned by the "march of intellect" —the physical world, or at least the world of physic, will come in for a share of revolution.

But to return. Our author has no hesitation in averring, that fevers of malarious origin are a very general cause of the chronic and common derangements of the digestive organs, to which so much attention has, of late years, been paid. In respect to the state of the pulse, Dr. M. justly observes that, "there are persons who cannot conceive a fever without an accelerated pulse; whereas, even in severe cases of remittent, the pulse often gives no indications of any disorder, or the very reverse of what such practitioners would have anticipated." The fact is, that in these forms of complaint, the pulse may be natural during the greater part of the day, but considerably accelerated for an hour or two, during the obscure paroxysm which marks the disorder. This often, perhaps generally, takes place in the evening or in the night, and consequently escapes the observation of the medical practitioner.

"The diseased state of mind may however exist at two distinct periods of the paroxysm, and under two different states of the pulse. Under the accelerated or contracted one, it is a state of peevishness or irritability, attended by the feeling of despondency or not: or it is a modification of the great leading passion anger, which together with fear, the equally inclusive and principal passion, forms those deranged states of mind appertaining to hypochondriasis, which appear under so many modes and modifications. And if under the full and slow pulse, it is commonly simple or passive despondency, or, in extreme cases, despair, so if the opposed condition or passion, irritability or peevishness, belongs to the accelerated pulse, that, in similarly extreme cases, may amount to anger, or to a tendency to that fundamental passion, easily excited by trivial causes; not seldom, difficult to restrain, even when no external cause is applied, or proceeding to causeless conduct, even in solitude, unaccountable to the patient himself."

This symptom of mental derangement may be the only one apparent to a bad observer. Despair and fear, especially the latter, appertain peculiarly to the class of marsh fevers—so much so that, in some parts of the Mediterranean, when these fevers are endemic, the only name by which they are known to the common people, is *scanto*—fear or fright. This circumstance is adduced by our author as corroborating his views of the connexion between hypochondriasis and malaria. He cautions the reader, however, against concluding that he is endeavouring to draw a sweeping conclusion that all cases of hypochondriasis are dependent originally on miasmal disorders. Dr. M. makes many judicious and ingenious observations on the effects of this slow or obscure remittent fever on the intellectual faculties, but we cannot so far disentangle them of the monstrous load of verbiage under which they lie buried, as to exhibit them here. The reader must plod, and labour, and unravel for himself. The two principal mental conditions induced in this way, are torpidity and excitement.

"The state of torpidity or inability accompanies that condition which must be considered as the cold stage, or which is the commencement of the diurnal paroxysm; being noticed, of course, only when the attack commences in the day and in the hours of labour, and therefore often passing without remark. And in every fever, this is the period of peculiar mental inability; the one observation confirming the other.

"The period of excitement, or of increased, if of hurried mental power, on the other hand, is the hot fit, or that which is here its substitute; a period of partial delirium: and here also, that condition of mind which is useful in moderation, is illustrated by the other, or by that excess which causes the imagination to run wild."

Other bodily symptoms are next investigated by Dr. M. Head-ache, though not always present, is much more common in women than in men. In the former, debility and head-ache often constitute the whole of the obvious disease. "With these symptoms alone, or apparently so, the disorder will sometimes run a course of six weeks, and with such severity as to confine the patient to bed." To the above symptoms may be added fits of restlessness or lassitude—occasional pains in the limbs—drowsiness, sometimes irresistible, and very remarkable in the chronic remittents of Italy and other malarious countries—natural sleep disturbed or irregular, being absent in the early part of the night, and coming on at the time when the patient ought to get up. The following is a curious instance of this class of disorders haunting an individual for 30 years!

"In this, the patient had, for thirty years, been subject to nearly all the diseases in rotation which I here rank under those of Malaria, namely, to remittent, to intermittent, and to almost every known variety of Neuralgia; having apparently acquired the incurable habit of these disorders at an early period of life. In several long intervals among those more marked ailments, the same person had also been affected, for long periods, with simple coma or drowsiness, and further with nocturnal awaking in the state of partial delirium just described: and it was his invariable remark, that the hour of awaking in this manner, was always precisely the same as that which marked the paroxysms of the intermittent and those of the Neuralgia, indicating their joint dependence on one cause and one habit; while that hour scarcely ever had varied by many minutes during the whole of his life of disease."

A disordered state of the digestive organs is so inseparably connected with the complaint in question, that our author seems doubtful whether to consider it a constituent part, or a consequence of the general malady resulting from malaria.

"Let physicians watch their own cases of this disease with this new light, and they will scarcely fail to find evidences of their own which will be much more satisfactory. And they will recollect also, when they reflect on their practice, how often they have found dyspepsia periodical under diurnal returns of various kinds, how often periodical and dependent on seasons, how often they have seen it cured by merely altering the hours of eating, how often by bark, or by arsenic, or by the other tonics that cure the remittent and intermittent diseases ; and how often by change of air, as also by mental affections, or causes operating on the imagination, such, among others, as the change of physicians ; all of them remedies for the intermitting diseases in question."

The author observes that, in the various works which have been written on dyspepsia, he has found no author who has taken this view of the complaint—who has looked to malaria as the cause. It is true that Dr. M. is original in making miasma so prominent a cause of dyspepsia, but if he will examine the works of authors again, he will find that "*impure air*"—which surely must be synonymous with *malaria*, has not been overlooked in the etiology of dyspepsia.* But malaria is only one of the thousand *physical* causes which disorder the digestive functions—and for one physical cause of this disorder, there are, at least, two moral causes ! It is but fair to say that Dr. M. considers the catalogue of real causes of dyspepsia too long for insertion in his work.

"Of the very numerous real causes of this common disease, I cannot pretend to give even a catalogue, as that would be to transgress my plan ; nor could I therefore enter upon any comparative view of the predominance of these several causes, or attempt to suggest what place the one here proposed may deserve among them."

Hysteria is considered as one of the symptoms attending this class of complaints—"though rather an incident than a portion of the disease." In some of the more strongly marked forms, however, of the low remittent, "the occurrence of the hysterical paroxysm, which is rarely more than a fit of crying, is commonly as regularly periodical as any other portion of the disease." Dr. M. has generally observed that it attends the subsidence of the pulse, or, in other words, the termination of the excitement. It is curious, that where the disease altogether is so slight, that the patient, if courageous, bears up against it, and does not complain, this hysterical weeping or feeling, is the only part of the disease which cannot be resisted. "A long-continued attempt at exertion, or at concealment which is exertion, is, in such cases, generally fol-

---

* While enumerating the *causes* of dyspepsia, Dr. Johnson makes the following remark :—"Where air, imbued with millions of *miasmata*, exhaled from every thing in the animal, vegetable and mineral kingdoms, is breathed, swallowed, and kept in contact with the skin, the effects are conspicuous in the sallow complexions, puny or capricious appetites, and *imperfect digestion* of the inhabitants."—*5th Ed*. p. 53.

lowed by a hysterical affection unusually severe." We coincide with Dr. M. in believing that most of those recorded cases of *periodical* hysteria belong to this class.

"It is one of the effects of this remittent, particularly when chronic or habitual, to produce those general derangements of the entire health which it would be tedious and equally useless to enumerate, as they are familiar to every one; while, with that, the temper, and even the moral character, as it may be considered, become also permanently or habitually injured."

Among the symptoms or consequences of the obscure malarious disorder now under consideration, we must not pass unnoticed those menstrual errors generally presented in the shape of amenorrhœa and dysmenorrhœa, the former particularly being often attended by a chlorosis that is mistaken for an original disease. We agree with our author that these complaints of the uterine system are "much more commonly the results of some derangement of the health than the sources of that ill-health by which they are accompanied."

Dr. M. has hazarded an opinion, that the climacteric disease, described by Sir Henry Halford, is a form of this chronic and obscure malarious malady. His arguments and illustrations will be found between page 105 and 108.

The original cause of this class of complaints, is, of course, the malaria so ably investigated in a preceding volume.

"Besides this original cause of all the evil, however, numerous other causes inducing debility will reproduce the relapse, and thus tend to perpetuate the disease; and the more readily as it is a more confirmed habit. Such are ordinary cold, fatigue, intoxication, bleeding, the excessive use of saline purgatives, mental affections, with others unnecessary to name; all of them equally efficacious in recalling the returns of a chronic tertian or quartan. Of all those causes, I would particularly notice here the use of purging, and mental affections; as, respecting the other, no one doubts much, and as they are commonly avoided. That what is called 'a course of salts' will very often reproduce an attack, I have abundant proof; and it is especially necessary to notice this, since it is commonly resorted to as a remedy for the imagined diseases to which the symptoms of this fever equally belong. Hence the frequently injurious effects of that fashionable folly, the frequenting of mineral wells; a practice resorted to by presumptuous patients, or by vulgar practitioners, as if it must be universally salutary, and was incapable of doing harm. And the common error in this case, as it is the especial cause of this erroneous and pernicious practice, is the mistaking the derangements which I formerly noticed for dyspepsia, as it is called, or liver complaints, or whatever else, under this received phraseology which is now so current; while the empirical practice to which I then alluded is applied without examination."

The existence of local pains in this class of complaints often induces to blood-letting, which generally aggravates the symptoms it was meant to relieve, besides protracting their duration.

"As to the influence of mental affections, it is rather a matter of curiosity than use, as the injurious occurrence of these can scarcely be guarded against; but it is, in the philosophy of physic, an interesting fact to observe, how instantaneously sudden grief, fear, disappointment, or other strongly depressing passions, will bring on that relapse which will generally run the same course as all the preceding."

Dr. M. remarks that, although this obvious disease has been so much overlooked, he has no doubt that it will shortly appear very common, now that it is distinctly pointed out. England has, till very lately, been so free from intermittent fevers (which form the readiest road to the analysis of the present disease) that but a few, proportionally, of medical men, have seen an ague at all. Hence, too, their attention has been but little directed to the investigation of malaria, as its source. There can be no doubt also, that in numerous instances, fevers resulting from miasmata have been confounded with typhus.

### Proximate Cause of Marsh Fever.

Our satirical author remarks that, it would be well if the professor, who spends months in exciting the wonder and applause of a juvenile audience with phraseology which he does not himself understand, would substitute for all this waste of words and time, the confession of his own ignorance. "For never yet has philosophy thriven by dressing up fiction and vain speculation in the garb of truth and sense. Physic knows not how the poison of fever acts, nor on what it acts—what are the preliminary effects which produce the symptoms that are obvious to our senses." We cannot, he says, even conjecture why these actions should cease—why they should be renewed—or why they should cease to be renewed. But, although Dr. M. can offer no theory of his own, he takes leave to criticise those of others. Dr. Clutterbuck's doctrine of cerebral inflammation he passes over. The action of malaria, say the French, is stimulant, and the symptoms of debility which succeed, are the effects of previous exhaustion. On this the following remark is made.

"If indeed the action of prussic acid, or lightning, or a cannon shot, is sthenic, then the assertion will not be disputed: and thus, he who, under the action of malaria, falls down instantaneously with apoplexy, has died of over-excitement."

The fever of marshes, say others, is a GASTRO-ENTERITE, or inflammation of the mucous membrane of the stomach or bowels—and every other effect and symptom is sympathetic or consequential—and the success of the practice is said to be confirmative of the theory. Dr. M. loudly protests against this exclusive theory; but he only adduces those arguments, which others, as well as ourselves, have repeatedly brought forward against the *abuse* of the doctrine. "Whatever dissections have taught, they have not taught us the cause of marsh fever." "They have taught us, that certain effects take place occasionally:—that is their use." The inflammations are effects not causes. Dr. M. admits the periodicity of inflammation in certain agues and neuralgias, but contends that these inflammations are not common, but specific inflammations; *sui generis*—and the circumstance of their being cured by bark and arsenic, proves them to be so. It is hardly necessary, after these observations, to say that our author gives up, in despair, any attempt at a proximate cause of the disease under review.

TREATMENT.

Dr. M. sets out by renouncing all idea of the treatment of remittent fever, as it appears in tropical climates, or in aggravated forms in any climate. "The most opposite opinions have been entertained, and the most opposite practices followed. As happens in tetanus, all these modes have failed—all these modes have seemed to succeed."

"The conclusion of him who knows nothing of physic, will probably be that the imagined remedies have had no concern in the cures, though he will scarcely conclude that they have had none in the ill success; while a fatalist in medicine, as fatalists there are, will perhaps determine that the efforts of the physician are nugatory as to either event."

Dr. M. touches on the administration of emetics, at the very beginning of a remittent, with the view of "putting it out." He does not seem to have much confidence in such a procedure, and thinks that it often produces or aggravates that peculiar inflammation of the stomach, which accompanies the fever. "How often death has been the result of such emetics, given improperly, or pushed too far, is well known." The general antiphlogistic measures necessary in the early stages of fever are passed over, as universally agreed upon. In respect to purgatives, Dr. M. makes some judicious observations. Where bleeding and antiphlogistic measures are proper, the saline purgatives, by producing watery motions, lessen the whole circulating mass—and are thus useful; but where the disease will not bear, or does not require sanguineous depletion, the said saline purgatives are improper, as tending to induce debility. Those resinous purgatives, however, which act locally, as wine, and merely promote the natural evacuations, are beneficial in all fevers, and almost all stages of fevers. On calomel, Dr. M. also makes some comments. In the remittents of hot climates, he has no reason to doubt its efficacy—in the milder fevers of our own country, he does not suppose that it exerts any specific influence "beyond such as is derived from its power over the biliary system." In regard to the chronic or relapsing variety, to which our author appears to have paid much attention, the following observations are deserving of notice, especially as they come from "one of those physicians who consider that this medicine (calomel) has been greatly abused."

"This remark is, that in the relapsing disorder, even if the attack should occur at the end of winter, in patients free from all suspicion of deranged liver or biliary affections, and when not the slightest indications of these can be traced, and when, further, the relapse may be the twentieth or fortieth to that patient, the operation of calomel is to produce obvious effects, which, if I need not specify them, physicians know well to be those which never occur except under derangement of this secretion. And at the same time, what is the important point here, it will be found that after every such effect of the medicine, the force of the disease diminishes, and that whenever the natural secretions recur, that particular relapse is about to terminate. Hence, therefore, I am led to consider, that even where it is least suspected, and indeed not to be believed present, there is often, in the chronic relapses, a derangement of the biliary functions; and that calomel, being the remedy for these, is apparently a remedy which cuts short or cures that relapse."

The use of the medicine is recommended as long as it is found to produce the evacuation of morbid bile:—when this disappears, the calomel becomes injurious.

On sudorifics, opium, cold affusion, and diet, some observations are made that do not require notice. Dr. M. very properly remarks, that food should never be given, however light, except in the intermissions or remissions. At all other times, even in the most mild and chronic remittent, it does much mischief by ruffling or irritating the stomach.

The grand point or question is that of administering tonics—and more especially bark, in this class of diseases. Dr. M. acknowledges that this is "a complicated question." There can be no doubt that there are instances where bark fails, or even aggravates the disease, even where no inflammatory symptoms are present. But, on the other hand, there are far more instances on record, where bark has cured the disease, during the actual and unequivocal existence of inflammatory action.

"It is as painful to a writer to leave his readers in suspense on points so essential as it would be presumptuous to decide; yet it may be suggested, that if, as will hereafter appear, the inflammatory affections of remittent are of a peculiar character, and not proper phlegmasiæ, and if certain visible and demonstrable ones are actually cured by this remedy and aggravated by evacuants, the question will not improbably be decided in favour of those who recommend it in all cases; and it may not be difficult then to discover that prejudice or incorrect observation will explain that testimony against it which has been thought to be derived from experience."

No doubt exists as to the utility of bark, where there are intermissions—or even pretty fair remissions, with general symptoms of debility. The very circumstance of there being a tendency to remission or intermission in a fever, is in favour of the utility of tonics, when not strongly contra-indicated by local phlogosis.

On the subject of wine, Dr. M. entertains some eccentric, or at least heterodox opinions. Is it proved, says he, that "wine increases inflammation, when existing, or produces a tendency to it in healthy subjects, or in diseased ones?" He asks whether these opinions are grounded on observation, or only hereditary dogmas "established no one knows why, and followed because they have been followed."

"But, granting that there are cases of inflammation, or a species of inflammation which wine would increase, physicians know full well that they are utterly ignorant of the real distinctions among inflammations which, to the sense or the eye, may appear the same; and that while there are some kinds or varieties which are to be cured by stimulants both local and general, as I shall hereafter show very fully, so are there inflammations, and apparently inflammatory states of the entire system with increase of circulation, where wine is a remedy instead of being injurious. Nor does it appear that the habitual use of wine produces a tendency to inflammation in healthy subjects; since it is notorious that among water-drinkers, the diseases of active inflammation are most frequent and require the most energetic treatment."

The remarks on blood-letting need not detain us. In robust subjects—at the first attack of the disease—the loss of blood is often useful in reducing that activity of the circulation, or that vigour generally, which renders the first portion of the paroxysm severe. When, also, there is unequivocal evidence of topical inflammation, we ought to have recourse to topical depletion. But, we need hardly say that Dr. M. is not among those physicians or surgeons who conceive that fever can be "put out" by venesection, however decisive, and however early employed,

"Were I indeed to indulge in that violence of generalization so usual with physicians, and so much too prevalent among those who undertake to point out or review a system of practice, it would be to assert that it were better that blood-letting should be utterly abolished in this fever, than that it should hold a place so egregiously abused."

From venesection Dr. M. makes a rapid transition to wine, the use of which in fever, says he, is "too often made a question of fashion and temper, rather than of rational and *sober* inquiry." How can we expect *sober* inquiry where wine is the topic? The following short quotation is all that is necessary on this point.

"That its singular combination of stimulant and sedative powers renders it one of the most convenient of the remedies generally classed under the vague term of tonics, seems to have been established by experience that can hardly be disputed; and the most determined theorist can scarcely deny to himself, that he has gained decided advantages from its use in the low or later stages of fever, and that it has often appeared to him the means of at least supporting the patient to a favourable termination."

Dr. M. after some remarks on subordinate agents in the treatment of fever, comes to the management of those milder varieties which he has described at such length in the volume before us. The following sarcastic observation is not destitute of foundation in truth—nor is it inapplicable to the *routine* practice of the day.

"A sweeping conclusion as to the ordinary simple fever of this character, would be, that it requires no remedies at all; and most assuredly, it is far better left to its own operations, or to nature, as the phrase is, than that it should be tampered with by intermeddling and routine practitioners. There is little good to be done by remedies; but it is not so as to the harm. Left to itself, its periods proceed in a very orderly manner to a favourable conclusion; but it is rarely so when a busy or active practice interferes. What the evils to be produced may be, can so easily be concluded from the preceding remarks, that I need not detail them."

A great deal of this routine and injurious polypharmacy depends more on the wretched and distracted *system* of medical practice and education in this country, than on erroneous views of disease. The medical man's *time* is his only fortune. Chronic diseases occupy far more of this valuable property than acute diseases; and as the general practitioner is not allowed to charge for his visits and his *advice*, he must necessarily send medicine in such proportion as will remunerate him for his *time*. Even when the physician or surgeon is called in, he must, as a matter of conscience, prescribe in the manner that repays the general practitioner for his attendance. He cannot, and indeed he ought not, to do otherwise—for, let it be remembered, that we are not to ruin ourselves for the sake of that public which persists in withholding from the profession the *proper mode* of remuneration. A patient will pay *only* for medicine—he considers the skill which directs that medicine as not deserving of any pecuniary remuneration. In God's name, then, let him have medicine—*usque ad nauseam*! The perversity of human nature, on this point, is really astonishing. The conscientious medical man, when he does not see clearly his way, and where no evident indication is to be fulfilled, would prescribe mere placebos—the common saline draught or camphor julep, which can do no harm, and by which he is remunerated for watching the disease. But the public is now got too enlightened to be put off with placebos. They must, forsooth, have medicines that will produce some ostensible *effect*, or no value is attached to the prescription! Assafœtida and camphor wont do. No! They must have something that will purge them, sweat them, make them sick, give or take away the appetite—in short, something that does violence to the constitution, or else the physician is an old woman, and the surgeon is picking their pockets by cramming down their throats a quantity of medicine, "which does them no good." Every medical man in *actual* practice—that is, who does not practise in the garret, with the grey-goose quill—can vouch for the truth of these observations. The natural and the inevitable consequence of such a state of things is, that all classes of medical practitioners are led into a system of prescribing *active* medicines, *in chronic diseases*, instead of managing the said complaints by a system of dietetics and general hygiene, that would be of far more use than medicine. Nothing but a general and consentaneous impulse and co-operation among medical practitioners can effect a change in this system, *at present;* though we are confident that reason and common sense will, sooner or later, correct the evil. A common *consent* or co-operation among medical men can hardly be expected, for some time to come, since the prevailing mania of the day is any thing but *concord*. Into whatever circle of society a medical man now goes, the common remark which he hears is—"what a *row* there is among the doctors!" In short, the medical profession is becoming the bye-word of derision among all the more enlightened classes, and we predict that the day is not far distant, when they will go in "sack-cloth and ashes," for their stupidity in being led away from the dignified paths of science into the arena of scurrility and personal abuse. But to return from this disgression.

Dr. M. does not mean to say that the disorder in question should be absolutely left to itself—he only cautions against heroic remedies. *Quietude* is considered an important measure.

"Thus when the whole visible paroxysm is nothing but a muscular debility which, with care and rest, would have been limited to a few hours, an exertion through that time will not only protract it through a considerable interval, but also produce other symptoms and greater inconveniences: such as, increase of pulse, headach, additional loss of appetite, and an augmented sense of general suffering. The same consequences also follow from mental exertion under the same circumstances; and if I need not repeat what I formerly said respecting sleep, it is plain that the reasoning is the same. In all these cases, that indulgence to the feelings or caprices of the patient which humanity ought to dictate, is also correct medical practice; much too often, however, controlled by the ascetic principle on one side, and by that of what, for want of a better term, I may call tyranny on the other."

The bowels should be kept as near a state of nature as possible, avoiding purgation, except where there are evident indications of morbid secretions or biliary derangement. "In this moderate fever, also, if wine is not absolutely necessary, it is always convenient or useful." There is one state, however, where active practice is necessary. It is by no means unusual for this anomalous disorder of health to take on a decidedly intermittent form, and then bark or arsenic is necessary, and is generally exhibited at once—thus terminating, in a few days, a series of teasing symptoms, or a condition of dubious convalescence, which might otherwise have dragged on for a long time. It is in this class of disorders that a sudden cure is often effected by change of habits, or rather change of air, especially where the original cause is malaria. Dr. M. asserts, and probably with some foundation in truth, that the routine practitioner not unfrequently hits on the real cure, without knowing what the disease is; and, while he is prescribing for mere debility, which is only a symptom or consequence of the disease, he gives the patient a certain number of bark draughts, for recruiting his strength, and this medicine strikes at the root of the disease, which is a veritable though obscure *remittent*. After giving vent to a severe philippic against writers and practitioners who have failed to remark (though we beg to say that they have not all failed to remark) the frequent termination of common continued fevers by an intermittent form, Dr. M. observes as follows:

"And if this particular fact, or the subsidence of a continuous fever to health through the intervention of an intermittent type, occurs very commonly in even the minor fevers, be their duration but a few days, or even one or two, then will it become additionally probable that even these fevers, be their technical names, or their imaginary causes, what they may, are dependent on the same cause as that which produces intermittents, or that they are true marsh or remittent fevers; since this is one of the essential and remarkable characters of remittent fever in its most unquestioned form. And if this particular mode of termination or evanescence never occurs in the fevers of contagion, or in the true typhus, which, from all my reading and observation is the fact, then is it at least proved, or rendered highly probable, that these minor fevers do not belong to typhus, however physicians may still determine to persist in referring them to the several doubtful or imaginary causes formerly discussed."

The great remedy, in all cases of obstinacy or relapse, is change of air, of habits, and of scene. Hence travelling, which combines all these, is equal to them all. In respect to medicine, the catalogue is small. The bowels should be regulated by the mildest means—the diet should be nutritious and plain—and light bitter tonics should be exhibited to improve the digestive process. These means would do more than all the farrago of drugs in Apothecaries' Hall.

We are so convinced of the fact, that a great number of disorders of the general health result from bad air, that we have taken great pains to concentrate Dr. Macculloch's views, and diffuse them widely through the profession. They are calculated to do much good, though some of them may have been carried too far. In succeeding articles we will take up the other subjects treated of in these volumes, and hope to render them still more interesting than the present—which, however, forms an indispensible preliminary to the investigation of malarious diseases generally.

---

### VII.

*Observations on the Peripneumonia of Children.* By Thomas Cuming, M.D. Assistant Physician to the Institution for Diseases of Children, &c.

[Dublin Transactions, vol. V.]

There are few, if any, diseases to which children are more liable, or which prove more fatal, if not timely remedied, than peripneumony. The situation which Dr. Cuming holds enables him to come before the public with ample materials resulting from personal observation, and of these materials we propose to exhibit the more important portions in this article.

In the dissections given by Cheyne and Hastings, the morbid appearances were chiefly confined to the lining membrane of the bronchia; whereas in almost every fatal case, our author found the parenchymatous and mucous tissues equally the seat of inflammatory action. Although the disease occurs at all ages, from a few days to eight or nine years, the most frequent subjects of attack, however, are children between nine months and two years—probably owing to the period of dentition. This disease occurring in children above two years of age, has generally been traced as a sequela of measles or hooping-cough. In some instances, the disease comes on very suddenly. The child goes to bed well, and rises next morning with all the symptoms of well-marked pneumonia. This, however, is rare.

"In general a trifling cough, with other slight symptoms of catarrh, precedes by a day or two the complete formation of the disease. When fully formed, the symptoms by which the disease is characterized are a hurried, laborious, and wheezing respiration; a frequent, short, and dry cough, and a greater or less degree of fever. With the fever are combined an extreme degree of restlessness and impatience, moaning, starting out of sleep, and aversion to be moved. The countenance, though
